# C/EBPβ regulates homeostatic and oncogenic gastric cell proliferation

**DOI:** 10.1007/s00109-016-1447-7

**Published:** 2016-08-13

**Authors:** Goncalo Regalo, Susann Förster, Carlos Resende, Bianca Bauer, Barbara Fleige, Wolfgang Kemmner, Peter M. Schlag, Thomas F. Meyer, José C. Machado, Achim Leutz

**Affiliations:** 1 Max-Delbrueck-Center for Molecular Medicine, Robert-Roessle-Str. 10, 13125 Berlin, Germany; 2 Humboldt-University of Berlin, Institute of Biology, 10115 Berlin, Germany; 3 Institute of Pathology and Molecular Immunology of the University of Porto, 4200-465 Porto, Portugal; 4 Max-Planck Institute for Infection Biology, 10117 Berlin, Germany; 5 Institut für Gewebediagnostik Berlin am MVZ des HELIOS Klinikum, 13125 Berlin, Germany; 6 Charité Comprehensive Cancer Centers, Charité-Universitätsmedizin, 10117 Berlin, Germany

**Keywords:** C/EBPβ, RUNX1t1, Gastric cancer, TFF1, Proliferation, Homeostasis

## Abstract

**Abstract:**

Cancer of the stomach is among the leading causes of death from cancer worldwide. The transcription factor C/EBPβ is frequently overexpressed in gastric cancer and associated with the suppression of the differentiation marker TFF1. We show that the murine C/EBPβ knockout stomach displays unbalanced homeostasis and reduced cell proliferation and that tumorigenesis of human gastric cancer xenograft is inhibited by knockdown of C/EBPβ. Cross-species comparison of gene expression profiles between C/EBPβ-deficient murine stomach and human gastric cancer revealed a subset of tumors with a C/EBPβ signature. Within this signature, the RUNX1t1 tumor suppressor transcript was down-regulated in 38 % of gastric tumor samples. The RUNX1t1 promoter was frequently hypermethylated and ectopic expression of RUNX1t1 in gastric cancer cells inhibited proliferation and enhanced TFF1 expression. These data suggest that the tumor suppressor activity of both RUNX1t1 and TFF1 are mechanistically connected to C/EBPβ and that cross-regulation between C/EBPβ-RUNX1t1-TFF1 plays an important role in gastric carcinogenesis.

**Key message:**

- C/EBPβ controls proliferation and differentiation balance in the stomach.
- Homeostatic differentiation/proliferation balance is altered in gastric cancer.
- RUNX1t1 is a C/EBPβ-associated tumor suppressor.
- RUNX1t1 negatively regulates C/EBPβ pro-oncogenic functions.

**Electronic supplementary material:**

The online version of this article (doi:10.1007/s00109-016-1447-7) contains supplementary material, which is available to authorized users.

## Introduction

Gastric cancer is one of the leading causes of cancer-related death in the developing world [1]. The oncogenic transformation of the gastric mucosa is often linked to persistent injury caused by chronic infection with *Helicobacter pylori* and chronic inflammation coincides with gastric cancer development [2]. The majority of sporadic gastric tumors belong to the intestinal type of gastric cancer, a histological entity characterized by expansive growth that retains a glandular structure. Despite the histological coherence however, no central common molecular pathway has been convincingly shown as aberrantly regulated in intestinal-type gastric cancer development. This is in contrast to another type of stomach cancer coined diffuse-type gastric cancer that is characterized by scattered growth and associated with loss of the adhesion protein E-cadherin [3–5].

Among the known common molecular changes in intestinal-type gastric cancer are enhanced expression of cyclooxygenase-2 (COX2) and diminished expression of the mucous-associated protein trefoil factor 1 (TFF1). Altered expression of both proteins is associated with cancer progression, although no recurrent mutations have been described [6–9]. Nevertheless, TFF1 knockout and COX2-overexpressing mice develop gastric tumors, highlighting the importance of the abnormal expression of these proteins for cancer development [10, 11]. Interestingly, CCAAT enhancer binding protein β (C/EBPβ) is also frequently overexpressed in intestinal-type gastric cancer and associated with both enhanced COX2 expression and loss of TFF1 [12, 13].

C/EBPβ is a transcription factor that belongs to the C/EBP family. C/EBPβ plays a central role in cell differentiation and cell lineage definition, as well as in inflammation control [14]. C/EBPβ has been implied to play a pro-oncogenic role in several other types of cancer, including mammary, skin, intestinal, and bladder cancer, as well as in acute myeloid leukemia (AML) and lymphoma [15–20]. C/EBPβ is thought to shield from apoptosis and to promote cell proliferation through several mechanisms, most notably in conjunction with cyclin D1 [14, 21–23]. Although C/EBPβ has not been reported as frequently mutated in tumorigenesis, signaling pathways regulating its activity and expression of its isoforms may account for a pro-oncogenic function of C/EBPβ [14, 24]. In gastric cancer, it is possible that C/EBPβ activation represents an upstream event with broader implications to tumorigenesis, of which TFF1 down-regulation and COX2 overexpression are hallmarks. Thus, a deeper insight into the role of C/EBPβ in normal and oncogenic stomach biology may help unraveling novel molecular candidates in gastric cancer development.

Here, we examined the functions of C/EBPβ in the murine stomach. Our results show that C/EBPβ controls the balance between proliferation and differentiation in the murine stomach. Cross-species analysis of gene expression between mouse C/EBPβ KO stomachs and human gastric cancer identified a C/EBPβ regulated gene signature in a subgroup of intestinal-type tumors. Within this signature, repression of RUNX1t1 stood out as a potential tumor suppressor event. Ectopic expression of RUNX1t1 reduced proliferation in gastric cancer cell lines and counteracted the repression of TFF1 by C/EBPβ. The RUNX1t1 promoter was found to be frequently hypermethylated in human gastric cancer cases. Our data suggest C/EBPβ activation and RUNX1t1 silencing as important events in the process of gastric carcinogenesis and suggests cross-regulation of C/EBPβ, TFF1, and RUNX1t1.

## Methods

### Human gastric cancer samples and microarray data

Human tissue samples were derived from patients that had undergone resection for sporadic gastric adenocarcinoma at the Robert-Roessle Clinic (1995–2003). The selection of samples, the procedure for histological classification and staging, the second blinded evaluation by an independent pathologist including assessment of tumor content in the pieces that RNA was extracted from as well as RNA extraction, and microarray procedure have been described elsewhere [25].

### Transgenic mice

C/EBPβ knockout (KO) animals were previously established in C57-Bl6 background [26]. Animals were bred and kept according to the institutional guidelines, and genotyped by PCR as previously described [26, 27].

### C/EBPβ knockdown cells and in vivo tumorigenic assay

MKN74 cells were infected with lentivirus containing GFP-tagged control shRNA and shRNA against C/EBPβ. Efficiency of knockdown was assessed by Western blot and proliferation was measured by BrdU incorporation assay. The effect of C/EBPβ expression on tumor formation was examined by subcutaneously implanting 3 × 10^6^ cells of both control MKN74 and ShRNA-mediated C/EBPβ-silenced MKN74 into 6–8-week-old male NIH(s) II-nu/nu nude mice, four mice per group. The animals were monitored weekly for tumor formation for 20 days after inoculation. Tumor sizes in two dimensions were measured with calipers, and volumes were calculated with the formula (*a* × *b*2) × 0.5, wherein “a” is the long axis and “b” is the short axis (in millimeters). Mice were maintained and sacrificed according to institutional guidelines, and at termination of the experiment tumors were excised, fixed, embedded, and analyzed by immunohistochemistry for Ki67 and C/EBPβ expression.

### Co-immunoprecipitation

Flag-tagged RUNX1t1 was expressed in MKN28 and MKN45 cell lines. Cells were harvested and lysed in buffer containing 50 mM Tris pH 8, 1 mM EDTA, 150 mM NaCl, 0.2 % NP-40, 5 mM MgCl_2_, 50 μM ZnCl_2_, and protease inhibitor cocktail (Roche®). Protein lysates were incubated at 4 °C with Protein A sepharose beads (Sigma®) for 1 h. Beads were then washed four times in lysis buffer and examined by Western blot analysis.

### Chromatin immunoprecipitation

Stomachs from two wild-type C57-Bl6 mice were excised, washed in ice-cold PBS, and incubated for 2 h in 30 mM EDTA. Epithelial cells were scrapped from the muscle layer and resuspended in 4 % paraformaldehyde for fixing and protein-DNA crosslinking. Cells were washed and resuspended in hypotonic buffer (2.5 mM Hepes, 1.5 mM MgCl_2_, 10 mM KCl, 0.1 % NP-40, 1 mM DTT, 0.5 mM PMSF) and incubated 10 min on ice for nuclei extraction. Nuclei were then resuspended in sonication buffer (50 mM Hepes, 140 mM NaCl, 1 mM EDTA, 1 % Triton X-100, 0.1 % Na-deoxycholate, 0.1 % SDS, 0.5 mM PMSF), incubated for 30 min, and sonified until 200–1000 bp fragments were obtained. Sonified chromatin was then immunoprecipitated with anti-C/EBPβ antibody (sc-150 Santa-Cruz Biotechnology®), or appropriate IgG control overnight at 4 °C, and 2 h incubation with Protein A Sepharose Beads (Sigma®). Beads were then washed in sonication buffer and TE buffer, and DNA eluted in elution buffer (50 mM Tris, pH 8.0, 1 mM SDS, 50 mM NaHCO_3_). DNA was de-crosslinked overnight at 65 °C and isolated by standard phenol/chloroform procedure. Binding of C/EBPβ to the TFF1 promoter was assessed by PCR (5′-gaaggtcatgtcaagggaggt-3′; 5′-atgagcttgcaccacgttct-3′). The promoter of the MUC5Ac was used as a negative control (5′-ctgtggagcatggggaaat-3′; 5′- gaaccacagacctgctccac-3′).

### Immunohistochemistry

Stomachs were obtained from 3-month-old C/EBPβ knockout (KO) mice animals. Stomachs were longitudinally excised, formalin-fixed, and embedded in paraffin. Gastric cancer tissue microarrays were obtained as described elsewhere [28].

Serial sections were obtained, deparaffinized, stained with hematoxylin and eosin, and examined by a pathologist. An additional group of sections were treated with 10 mM citrate buffer and stained with 1:100 anti-Ki67 (MIB1 DAKO), 1:500 anti-C/EBPβ,1:50 anti-TFF1 (sc-150 and sc-517213 Santa Cruz Biotechnology), or 1:500 anti-RUNX1t1 (SAB2102065 Sigma®) antibody. After washing with PBS with 0.02 % Tween and incubation with horseradish peroxidase-bound secondary antibody (GE Healthcare®), development was performed using di-amido-benzidine.

### BrdU assay

Cells with stable C/EBPβ knockdown were sorted and plated to 40 % confluence. Cells were also transfected with RUNX1t1 and analyzed for BrdU incorporation after 48 h. Briefly, cells were incubated with 1 M Bromo-deoxy-uridine for 20 min and then trypsinized and harvested in ice-cold PBS. Cells were then fixed, permeabilized, and stained with fluorescent anti-BrdU antibody according to the APC-BrdU flow kit protocol (BD Biosciences®). Dead cells were stained with 7-AAD and BrdU-positivity was then assessed by flow cytometry.

### Electrophoretic mobility shift assay (EMSA)

MKN28 and MKN45 cells were transfected with increasing amounts of RUNX1t1. Nuclear extracts were prepared from transfected cells, quantified, and incubated with radioactively labeled (32P) palindromic C/EBPβ binding oligonucleotide. EMSA was performed as previously described [29]. Anti-C/EBPβ antibody (sc-150 Santa-Cruz Biotechnology®) was added to super-shift samples and competition with 50-fold excess of unlabeled oligonucleotide to confirm specificity.

### Total RNA extraction, cDNA synthesis, and quantitative real-time PCR

For RNA extraction from mouse tissue, stomach sections were frozen in liquid nitrogen after excision and finely ground in a mortar. For RNA extraction from gastric cancer cells, these were harvested in ice-cold PBS and pelleted at 2000 rpm. Lysis buffer was then added to the obtained powder or to the pellet which was then vigorously resuspended using a 3-ml syringe. RNA was extracted using a universal RNA extraction kit (Roboklon®). RNA was quantified, cDNA synthesized by standard methods, and SYBER green quantitative real-time PCR performed (see Supplementary Table 4 for primer sequences).

### Plasmids

For the construction of C/EBPβ isoform expression vectors, LAP*, LAP, and LIP were cloned from human cDNA by PCR, following digestion with restriction enzymes, ligation into pcDNA3-flagged plasmid, and ampicillin selection. RUNX1t1 expression plasmid (pCMV-3xFlag-ETO) was obtained from ADDGENE® (ref: #12507). TFF1-luciferase reporter plasmid was similarly cloned from human cDNA into a pGL3-basic plasmid. For the construction of C/EBPβ knockdown vectors, shRNA (5′-gccgcgacaaggccaagatgc-3′) was inserted into a pLVTH-M lentivral vector.

### Tissue culture, transfection, and luciferase assay

MKN28, MKN45, and MKN74 cell lines were grown in RPMI medium (Gibco®). For transfection, cells were trypsinized, seeded, and grown to 50–60 % confluence. C/EBPβ isoform plasmids and/or RUNX1t1 plasmid were resuspended in serum-free medium with transIT (Myrus®) transfection reagent and added to the cells. Protein and RNA were extracted after 48 h and analyzed by Western blot and real-time PCR. For Luciferase assay, expression plasmids were co-transfected with TFF1-luciferase reporter plasmid and normalization MCV-Renilla plasmid. After 48 h, cells were lysed and reporter activity measured in a luminometer. Results were presented as a Luciferase/renilla activity ratio.

### RUNX1T1 promoter methylation analysis

Methylation analysis of the RUNX1t1 promoter was determined by methylation-specific PCR (MSP), as previously described [30]. MSP method distinguishes unmethylated from methylated alleles in a given gene based on sequence changes produced after bisulfite treatment of DNA, which converts unmethylated but not methylated cytosines to uracil. Subsequently, PCR using primers specific to either methylated or unmethylated DNA was performed. Genomic DNA (350 ng) was bisulfite-treated and purified with EZ DNA Methylation Kit Gold (Zymo Research, CA, USA®). The primer sequences of RUNX1t1, for both methylated and unmethylated reactions, were as previously described [30]. One hundred nanograms of bisulfite-modified DNA was used in each PCR. Amplification was carried out for 36 cycles (30 s at 95 °C, 30 s at 56 °C, and then 30 s at 72 °C). Control PCRs lacking genomic DNA were performed for each set of reactions. Amplified products were separated by electrophoresis in a 2.5 % agarose gel.

### Bioinformatic microarray data analysis and statistical analysis

The raw data files (.text files for murine Agilent Technologies® arrays and .cel files for human Affymetrix GeneChips®) were imported into GeneSpring GX 12.1 software (Agilent Technologies®) as two separate species-specific experiments. All subsequent microarray data analyses were performed using this software. Preprocessing (background correction, normalization, and probe summarization) was performed according to the RMA algorithm followed by baseline transformation to the median of all samples (in one experiment). Quality control was done by assessment of inter-array correlation analysis calculating the correlation coefficient of each array to every other one. By this means, one array of the murine gene expression experiment was identified to show relatively weak correlation to most of the other samples and thus excluded from further analysis. The human arrays yielded correlation coefficients between 0.829 and 0.972, with an arithmetic mean of 0.917 and the murine arrays between 0.991 and 0.924 with a mean of 0.9. In the murine array experiment, only probes owning “detected” flags in at least three arrays (34,150 probes) were used for further analyses. Genes whose expression between groups of samples was significantly different were identified by Welch test with *p* ≤ 0.01 being the significance cutoff. The fold change (FC) of expression between groups was calculated as the fold difference between group means. Gene annotation information was obtained from GeneSpring GX software (state of 08/2012). For hierarchical clustering, “Euclidean distance” and “complete linkage” were used as distance metric and linkage algorithm. The migration of genes between the murine and human microarray experiment was performed using the Orthology Search Tool of bioDBnet athttp://biodbnet.abcc.ncifcrf.gov/.

## Results

### C/EBPβ knockdown reduces the tumorigenic potential of gastric cancer cells

**Fig. 1:**
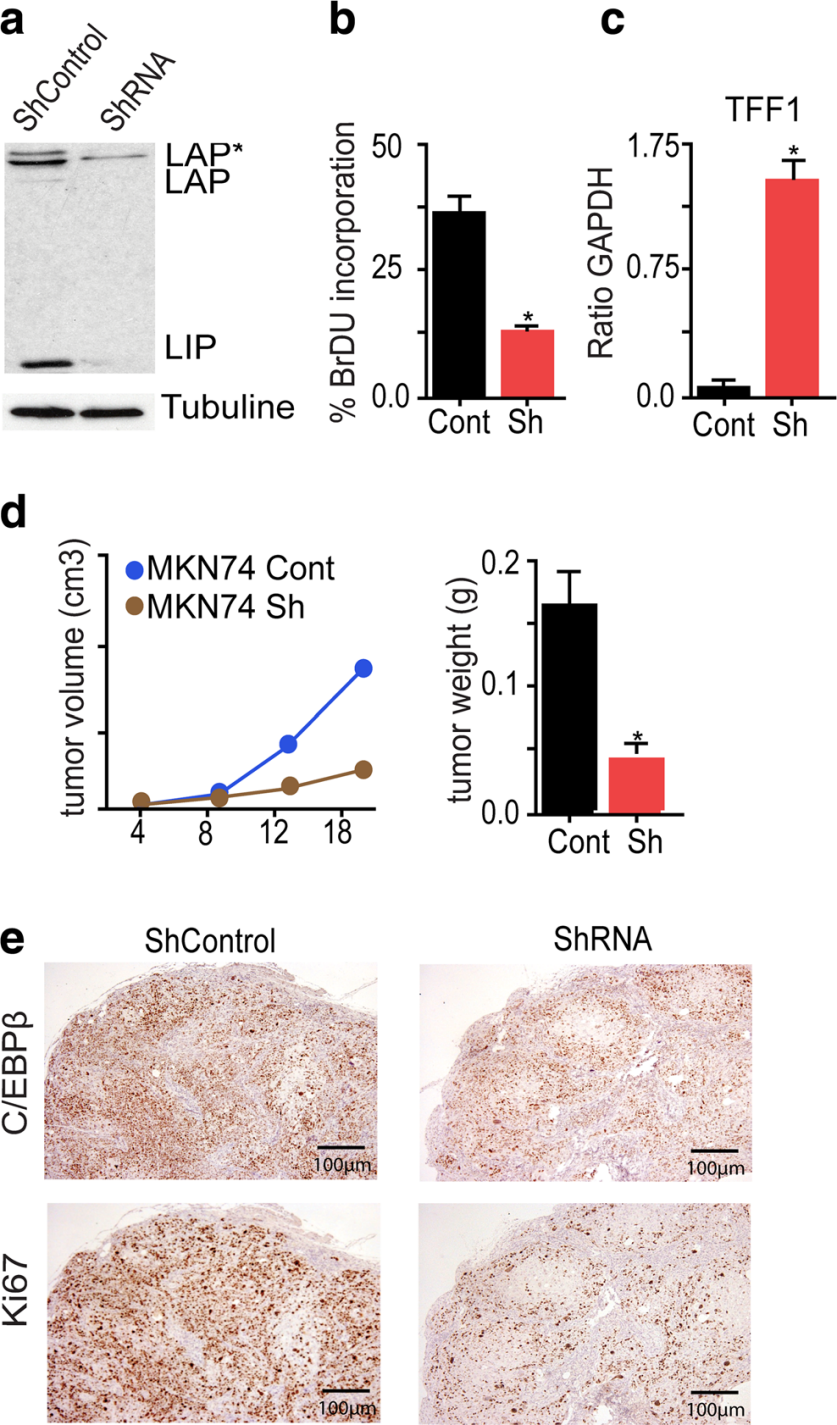
C/EBPβ controls gastric cancer cell proliferation. **a** Stable knockdown (KO) of C/EBPβ in MKN74 cell line evaluated by protein blotting. **b** Cell proliferation was determined by BrdU analysis. Cells were labeled with BrdU and incorporation was determined by flow cytometry (FACS) and plotted against 7-AAD-positive cells, as a measure of DNA content. **c** Expression of the gastric differentiation marker TFF1 was assessed on the stably transfected cells. Expression was increased in MKN74. **d** Equal amounts of control and stable C/EBPβ KO MKN74 cells were injected into nude mice and tumor volume and weight was assessed at different time points. Tumors originated from C/EBPβ KO cells were smaller than tumors in the controls. **e** Ki67 staining revealed reduction of proliferation in the KO-derived tumors. All bar graphs represent the result of at least three independent measurements; *asterisk* indicates *p* < 0.05

Enhanced C/EBPβ expression mainly in the human intestinal-type gastric cancer subtype has previously been observed [12, 13]. The functional importance of high C/EBPβ expression in gastric cancer was examined by stable knockdown in a human gastric cancer cell line using a viral-based GFP-tagged short hairpin RNA. C/EBPβ knockdown efficiency in MKN74 was approximately 70 %, as confirmed by protein immunoblotting (Fig. 1a). Proliferation was examined by BrdU incorporation and, as shown in Fig. 1b, proliferation was reduced after C/EBPβ knockdown. C/EBPβ has previously been reported to repress the gastric differentiation marker and tumor suppressor TFF1 [31, 32], and in accordance with these data, C/EBPβ knockdown enhanced TFF1 expression in MKN74 (Fig. 1c).

The tumorigenic potential of MKN74 before and after C/EBPβ knockdown was compared by xeno-transplantation in immune-compromised mice, as shown in Fig. 1d. Twenty days post-injection, C/EBPβ knockdown cells formed markedly smaller tumors than parental cells, with less weight and volume (Fig. 1c). Ki67 staining showed reduction of cell proliferation in tumors originating from C/EBPβ knockdown cells in comparison to controls (Fig. 1d). Interestingly, re-expression of C/EBPβ was accompanied by proliferation in tumors. Consistently, knockdown cells in tissue culture required frequent sorting to prevent overgrowth of cells that regained C/EBPβ expression, suggesting selection for C/EBPβ re-expression as a growth advantage for gastric tumor cells. Taken together, these results suggest that C/EBPβ plays an important role in gastric cancer cell proliferation.

### C/EBPβ knockout mice display imbalanced differentiation/proliferation of the gastric mucosa

**Fig. 2:**
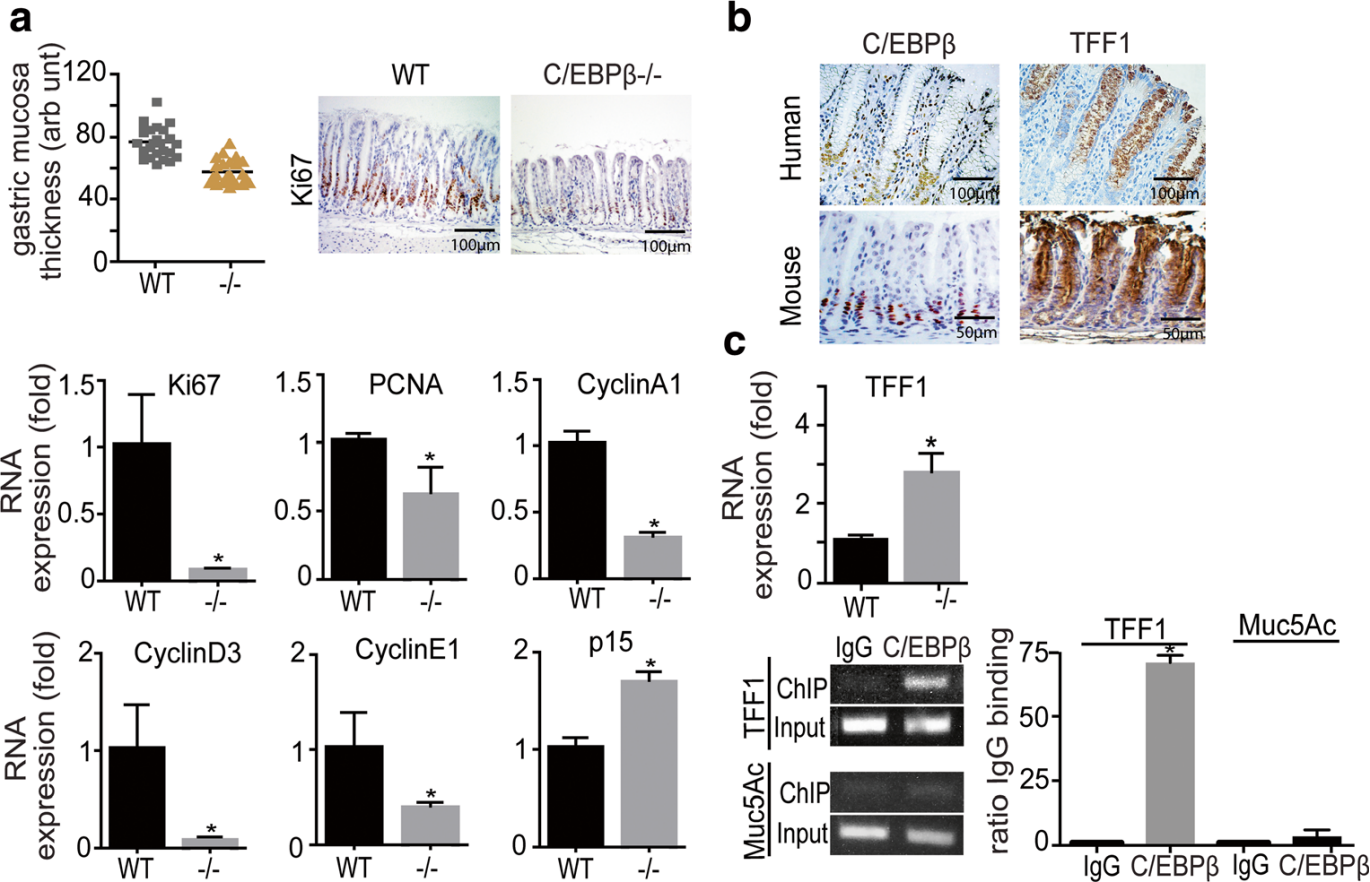
Analysis of the gastric phenotype of the C/EBPβ knockout (KO) mouse. **a** Quantification of the C/EBPβ KO mice and WT antral gastric mucosa thickness (in arbitrary units). Adjacent immunohistochemical panel depicts the reduction of Ki67-positive cells in the C/EBPβ KO mucosa. *Lower panels* show qPCR evaluation of Ki67, PCNA, Cyclin A1, D3, E1, and p15 in the gastric mucosa of WT and C/EBPβ KO mouse stomach (five animals/group, 3 months old). Expression values were first normalized to GAPDH expression and values are presented as fold of WT expression. **b** Mutually exclusive expression of TFF1 and C/EBPβ in the normal human (*upper panel*) and mouse (*lower panel*) stomach epithelium; C/EBPβ is expressed in proliferative cells of the neck zone and TFF1 in differentiated mucous epithelium. **c** Increased expression of mRNA of differentiation protein TFF1, in the C/EBPβ KO mouse mucosa as measured by qPCR. *Lower panel* show ChIP assay on disaggregated wt mouse stomach cells, showing in vivo binding of C/EBPβ to the TFF1 promoter both on an agarose gel (*left*) and by qPCR quantification. Results are presented as ratio to anti-IgG control binding. Binding to the Muc5ac promoter was used as a negative control. All bar graphs represent the result of at least three independent measurements; *asterisk* indicates *p* < 0.005

Analysis of nullizygous C/EBPβ stomachs (*n* = 5) revealed a significant (*p* < 0.001) reduction in the thickness of the antral gastric mucosa and diminished numbers of Ki67-positive cells, as compared to the wildtype (WT) (*n* = 8). No other histological abnormalities were observed and the corpus region from knockout animals was largely indistinguishable from the WT. To gain further insight into the causes of reduced mucosal thickness, expression of cell cycle-related genes was examined. As shown in Fig. 2c, reduction of Ki67 and of proliferating cell nuclear antigen (PCNA) in the KO antral mucosa was evident by quantitative PCR (qPCR) in accordance with histological observations. Furthermore, expression of the CDK inhibitor p15 was increased and expression of cyclin A1, cyclin D3, and cyclin E1 were reduced.

Similarly to human gastric mucosa, TFF1 was not expressed in proliferating cells of the neck zone in murine WT gastric epithelium, and expression of C/EBPβ and TFF1 was found to be mutually exclusive (Fig. 2b). Real-time PCR confirmed the increased expression of TFF1 in C/EBPβ KO mucosa (Fig. 2c). Chromatin immunoprecipitation (ChIP) with anti-C/EBPβ antibody in disaggregated murine stomach epithelial cells showed that C/EBPβ binds to the promoter of TFF1 in vivo (Fig. 2c). Taken together, the data suggest a role of C/EBPβ-mediated repression of gastric differentiation in proliferating cells of the normal gastric mucosa.

### Cross-species gene expression profiling reveals a subset of intestinal-type gastric tumors with a C/EBPβ regulated signature

The apparent similarities between human and murine gastric C/EBPβ biology raised the question whether the homeostatic and oncogenic C/EBPβ-dependent proliferation share common molecular mechanisms. We therefore compared the gene expression profiles derived from C/EBPβ KO mice with previously analyzed human gastric adenocarcinoma samples. These samples were isolated under the supervision of a pathologist, and areas enriched for epithelial tumor cells were selected [25].

Differentially expressed genes between the C/EBPβ KO (*n* = 5) and WT (*n* = 4) mice were identified by Welch test. Significance in differential expression was accepted at *p* ≤ 0.01 and a FC of larger than >1.5. These cutoff criteria yielded 171/25 annotated/non-annotated unique transcripts (represented in 233 probes) as up-regulated in the C/EBPβ KO and 79/12 annotated/non-annotated unique transcripts (represented in 135 probes) as down-regulated (Supplementary Tables 1 and 2 show the 20 most significantly regulated genes).

**Fig. 3:**
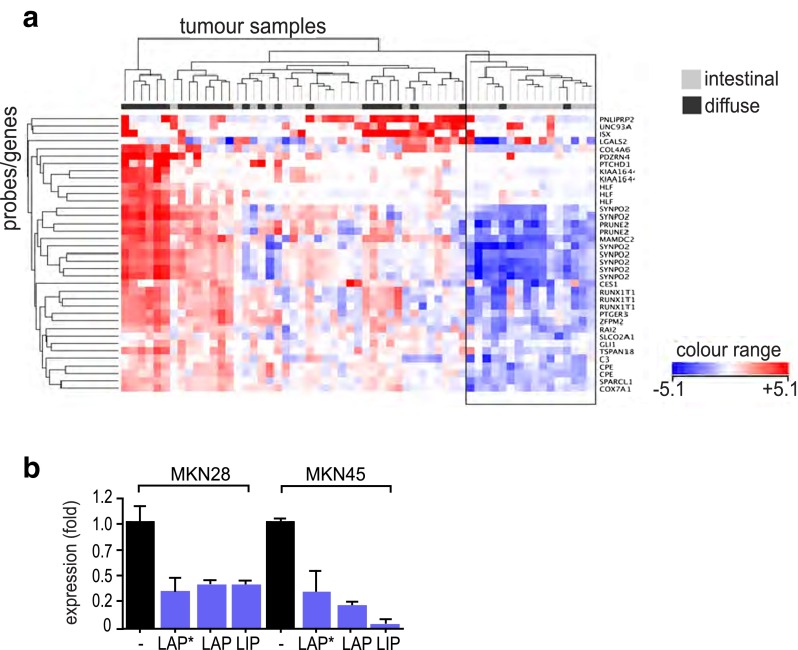
**a** Cross-species comparison of gene expression. Two-way hierarchical clustering was performed on the human gastric cancer samples using a strongly regulated gene cluster (shown in Supplementary Fig. 2) from microarray-derived genes that differed between murine C/EBPβ KO and WT stomach (*p* ≤ 0.01, FC ≥ 1.5). Depicted are the resultant gene and sample dendrograms and the corresponding expression intensity heatmap. The *black box* indicates a tumor cluster in which most of the genes show down-regulation (*bluish spots*). This tumor group consisted of 16 of the original 59 (≈27 %) samples and contained primarily cancers of the intestinal histological type. **b** Transfection of C/EBPβ isoforms LAP*, LAP, and LIP into gastric cell lines MKN28 and MKN45 repressed RUNX1t1 expression as measured by quantitative PCR. All bar graphs represent the result of at least three independent measurements; *asterisk* indicates *p* < 0.001

Next, the combined list of up- and down-regulated genes (FC > 1.5, *p* ≤ 0.01) derived from the murine C/EBPβ KO profiling data was used to cluster data obtained from human gastric cancer microarrays. The resulting gene expression heatmap revealed a group of genes that showed explicit regulation (indicated by dark bluish/reddish spots in the heatmap) across the human cancer samples (Supplementary Fig. 1, C/EBPβ regulated gene cluster, indicated by box), among a majority of genes that did not show any overt deregulation (whitish spots in heatmap). Genes contained in the strongly regulated subset were then used alone to re-cluster all human cancer samples. The resultant dendrogram and expression heatmap (Fig. 3a) revealed a group of cancer samples (Fig. 3a, black box) that exhibit down-regulation of the majority of these genes. The group consisted of 16 of the original 59 (≈27 %) samples and contained primarily cancers of the intestinal histological type. Importantly, most of the down-regulated genes in this cancer subgroup are up-regulated in the C/EBPβ KO gastric mucosa (changes ranging from 1.5- to 2.3-fold; Table 1), clearly identifying them as C/EBPβ repressed genes.

**Table 1:** Genes from the C/EBPβ-clustered intestinal-type gastric cancer genes, showing their regulation in both intestinal-type tumors and C/EBPβ KO stomachs. Down-regulated genes in intestinal-type gastric cancer are up-regulated in the C/EBPβ KO stomach

	Gene name	*p*	Regulated in	FC	*p*	Regulated in	FC
		(int. vs. diff.)	intestinal		(C/EBPβKO vs. WT)	C/EBPβ	
			GC			KO	
COL4A6	Collagen, type IV, alpha 6	0.026065	Down	2.2	0.003434	Up	1.8
COX7A1	Cytochrome c oxidase,	1.34E−07	Down	2.6	0.008941	Up	2.2
	Subunit VIIa 1						
CPE	Carboxypeptidase E	8.69E−04	Down	2	8.96E−04	Up	1.6
GLI1	GLI-Kruppel family member GLI1	3.39E−05	Down	1.9	0.001579	Up	1.6
HLF	Hepatic leukemia factor	9.51E−04	Down	2.3	0.007635	Up	1.6
MAMDC2	MAM domain containing 2	1.44E−06	Down	6.2	0.007404	Up	2.3
PDZRN4	PDZ domain containing RING finger 4	0.005077	Down	2.9	0.007338	Up	2
PTCHD1	Patched domain containing 1	0.020572	Down	2.1	0.00189	Up	1.5
PTGER3	Prostaglandin E receptor 3	1.89E−07	Down	3.7	0.004076	Up	1.6
	(subtype EP3)						
RAI2	Retinoic acid induced 2	6.97E−05	Down	2.6	0.008717	Up	1.6
RUNX1T1	runt-related transcription factor 1; translocated to, 1 (cyclin D-related)	1.32E−08	Down	5.2	0.00741	Up	1.5
SPARCL1	SPARC-like 1	9.97E−10	Down	2.7	0.007544	Up	1.5
ZFMP2/FOG2	Zinc finger protein, multitype 2	9.81E−08	Down	4.4	0.001298	Up	1.6

In order to validate the results obtained by microarray comparison, we selected three C/EBPβ repressed genes, FOG2, SPARCL1, and RUNX1t1, and analyzed their expression by qPCR. Examination of WT and C/EBPβ KO stomach confirmed up-regulation of these genes in the gastric mucosa of C/EBPβ KO mice (five animals/group; Supplementary Fig. 2A). It was also important to examine the expression of FOG2, SPARCL1, and RUNX1t1 in normal human gastric mucosa, as no matching normal tissue samples were available from the initial human gastric cancer microarray analysis [25]. As shown in Supplementary Fig. 2B, expression of all three genes was down-regulated in intestinal-type gastric cancer in comparison to normal tissue; however, a subset of diffuse-type tumors overexpressed RUNX1t1, compliant with a different etiology of these tumors. The microarray data-derived expression of C/EBPβ and RUNX1t1 in all analyzed 59 human gastric cancer samples is depicted in Supplementary Fig. 3.

### RUNX1t1 plays a tumor suppressive role in human gastric cancer and modulates C/EBPβ activity

A connection between RUNX1t1 and C/EBPβ has previously been suggested to control the proliferative clonal expansion phase in adipogenesis [33]. Indeed, the C/EBPβ KO stomach has markedly increased levels of RUNX1t1, and ectopic expression of C/EBPβ isoforms (LAP*, LAP, and LIP) in MKN28 and MKN45 cell lines inhibited RUNX1t1 expression (Fig. 3b), suggesting C/EBPβ mediated repression of RUNX1t1.

**Fig. 4:**
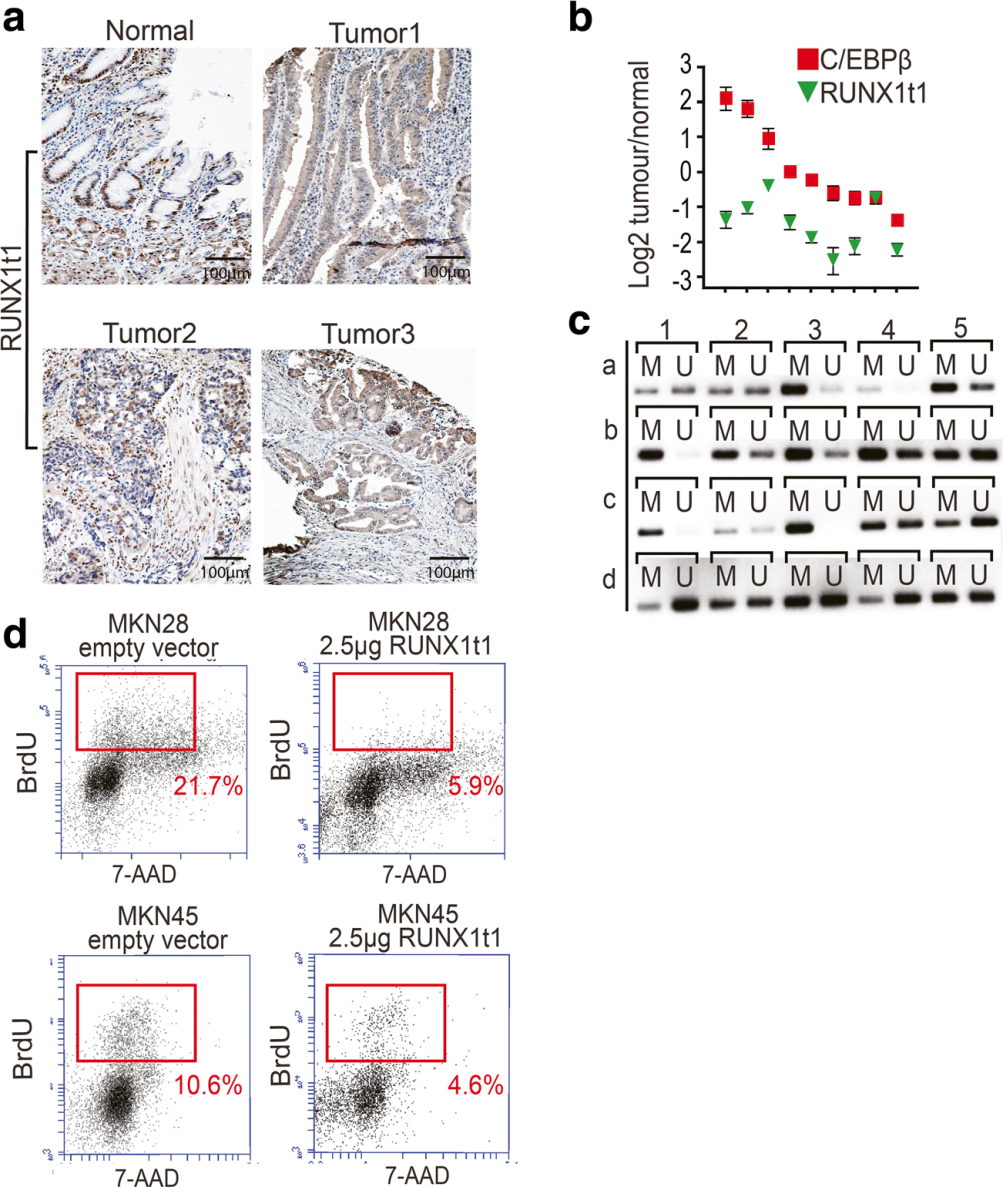
RUNX1t1 and gastric cancer. **a** RUNX1t1 expression was evaluated by immunohistochemistry in 64 human gastric cancer samples, and staining was classified by comparison to the expression in the normal mucosa (*left panel*). Thirty-eight percent of the cases showed reduced expression of nuclear RUNX1t1 (Tumor 1–3) in comparison to staining in the normal epithelium. **b** In 10 gastric tumors with reduced RUNX1t1, RNA levels were examined for C/EBPβ expression by qPCR. Only 3 out of 10 cases showed higher C/EBPβ expression as compared to WT. **c** The methylation status of the RUNX1t1 promoter was evaluated by methylation-specific PCR. Bisulfite treatment of tumor DNA converts unmethylated but not methylated cytosines to uracil, and subsequent methylation-specific PCR detects either methylated (*M*) or unmethylated (*U*) DNA. Fifty percent of the analyzed human gastric cancer cases (rows a-b, columns 1–5) present RUNX1t1 promoter hypermethylation. An increase in the methylation status is considered when the PCR product with methylation-specific primers is more intense than the one produced by non-methylated specific primers. **d** Ectopic expression of RUNX1t1 in MKN28 and MKN45 gastric cancer cell lines reduces gastric cancer cell proliferation as measured by BrdU incorporation assay. S-phase percentages are indicated in the FACS plots

Expression of RUNX1t1 protein was evaluated by tissue microarray immunohistochemistry on 64 human gastric cancer samples. Nuclear staining was classified as strong, moderate, weak, or absent, referencing to RUNX1t1 expression in the normal mucosa (classified as moderate). As shown in Fig. 4a, 25 out of 64 (38 %) tumor samples showed weak or absent RUNX1t1 protein staining. To further assess the connection between C/EBPβ and down-regulation of RUNX1t1 in gastric tumors, we selected tumor-RNAs showing reduced levels of RUNX1t1. In 3 out of 10 cases, we found a convincing inverse correlation between low RUNX1t1 and high C/EBPβ expression (Fig. 4b); however, the data may also suggest alternative routes of RUNX1t1 down-regulation in gastric cancer. Sequencing of RUNX1t1 from 26 gastric cancer patients failed to disclose mutations that would explain loss of RUNX1t1 protein (data not shown); however, analysis of the RUNX1t1 promoter revealed hypermethylation in 10 out of 20 gastric cancer DNA samples (Fig. 4c).

In order to determine whether RUNX1t1 down-regulation in gastric cancer cells has functional consequences on cell multiplication, we rescued RUNX1t1 expression and evaluated proliferation by BrdU incorporation. As shown in Fig. 4d, FACS analysis of BrdU-positive cells showed that re-expression of RUNX1t1 led to a significant decrease in cell proliferation.

### RUNX1T1 interacts with C/EBPβ to abolish DNA binding and TFF1 promoter repression in stomach cells

**Fig. 5:**
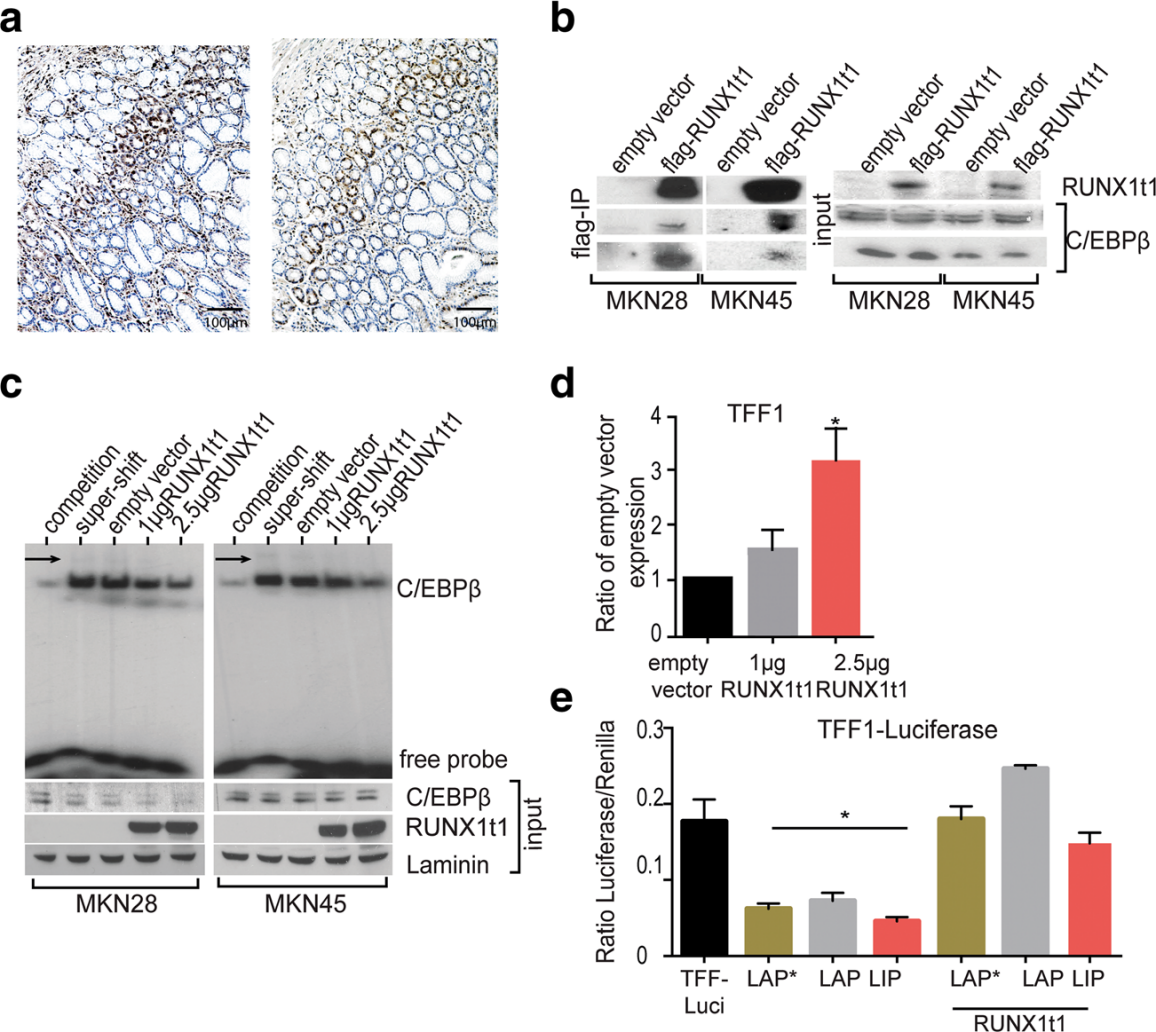
RUNX1t1 modulates C/EBPβ activity. **a** Immunostaining shows C/EBPβ and RUNX1t1 colocalization in the neck zone of the normal human gastric mucosa. **b** Flag-Immunoprecipitation after transfection of gastric cell lines with a flag-tagged RUNX1t1 pulls down C/EBPβ. Visible in the input Western blot is also that RUNX1t1 does not affect C/EBPβ expression. **c** EMSA using a radiolabeled C/EBPβ consensus probe shows that transfection of RUNX1t1 to gastric cancer cells reduces the binding of C/EBPβ to DNA in MKN28 and MKN45 cell lines. *Arrow* indicated the super-shift. Also visible in the control Western blots, RUNX1t1 has no effect on basal C/EBPβ expression. **d** A RUNX1t1-dependent increase in the expression of TFF1 was visible by real-time PCR in the MKN74 cell line. **e** Luciferase assay in MKN45 cells transfected with the TFF1-luciferase fused promoter shows that co-transfection with RUNX1t1 reverts the repressive potential of C/EBPβ on the TFF1 promoter

Immunohistochemical analysis of normal human stomach showed that RUNX1t1 and C/EBPβ co-localize to the proliferative neck zone of the normal gastric mucosa (Fig. 5a). In order to analyze if co-expression also entails physical interaction, we expressed Flag-tagged RUNX1t1 in MKN28 and MKN45 cell lines, where basal RUNX1t1 expression is very low. As shown in Fig. 5b, all endogenously expressed C/EBPβ isoforms in both gastric cancer cell lines were co-precipitated with RUNX1t1.

Previously it had been shown that RUNX1t1 inhibits the DNA binding of C/EBPβ in pre-adipocytes [33]. EMSA with nuclear extracts from MKN28 and MKN45 gastric cancer cell lines transfected with increasing amounts of RUNX1t1 showed that RUNX1t1 caused a dose-dependent decrease of C/EBPβ binding to its DNA consensus sequence. RUNX1t1 did not significantly alter nuclear C/EBPβ expression levels (Fig. 5c). As C/EBPβ represses TFF1 expression, RUNX1t1 may release C/EBPβ or inhibit its binding to DNA and thus enhance TFF1 repression. In accordance with this notion, MKN74 cells showed a dose-dependent increase in TFF1 expression after ectopic expression of RUNX1t1 (Fig. 5d). Likewise, co-transfection of RUNX1t1 and C/EBPβ, together with a TFF1 luciferase reporter abolished the repression of the TFF1 promoter in the MKN45 by C/EBPβ (Fig. 5e).

## Discussion

Comparison of gene expression profiles from C/EBPβ KO mice and human gastric cancer samples provided an insight in C/EBPβ-related molecular mechanisms. Taken together, our data suggest a tumorigenic function of C/EBPβ in the development of a subset of gastric tumors.

Data show that the function of C/EBPβ in gastric cancer is embedded in the homeostatic regulation of the gastric mucosa. Absence of C/EBPβ from the murine stomach shifts the balance from epithelial proliferation towards differentiation. Although this was primarily observed in the thinner antral epithelium, we do not exclude potential C/EBPβ-dependent effects also in the proximal stomach, although they may be more difficult to observe due to the more complex architecture of the tissue. Deregulation of pathways that sustain C/EBPβ functions, such as inflammatory signals, may enhance proliferation and repression of differentiation genes, such as TFF1, that ultimately promotes tumor development [11]. The data also show that C/EBPβ is mandatory for the tumorigenic potential of gastric cancer cell lines by promoting cell proliferation and confirm the repressive effect of C/EBPβ on the expression of TFF1.

Expression profiling data of human gastric cancer samples and comparison with C/EBPβ KO mouse-derived expression data identified a subset of tumors with a C/EBPβ-regulated signature. These tumors mostly belong to the intestinal type or may define a novel subtype. Despite the absence of TFF1 from the gene list, one of the de-regulated cluster genes, RUNX1t1, has previously been connected to both gastrointestinal abnormalities [34] and to suppression of C/EBPβ functions [33].

RUNX1t1, also known as MTG8 or ETO, is the recurrent t(8;21) translocation partner of the AML-ETO (RUNX1/MTG8) fusion protein. AML-ETO accounts for 15 % of acute myeloid leukemia and 40 % of M2-type leukemia, probably by interference with the differentiation inducing functions of C/EBPα and PU.1 [35, 36]. RUNX1t1 is also a candidate tumor suppressor in ovarian cancer [30] and loss of RUNX1t1 expression has been associated with metastasis in pancreatic cancer [37]. Down-regulation of RUNX1t1 during homeostasis and in intestinal-type gastric cancer may initially occur through C/EBPβ; however, analysis of DNA methylation showed that the RUNX1t1 promoter was frequently methylated in human gastric cancer samples. RUNX1t1 promoter hypermethylation has also been observed in ovarian cancer [30] and thus suggests an alternative route of RUNX1t1 gene silencing in carcinogenesis.

Our data suggest that the RUNX1t1 tumor suppressive function might be related to repression of C/EBPβ DNA binding, reminiscent to its function in the adipogenic clonal expansion phase that requires expression of C/EBPβ but also RUNX1t1 to prevent premature induction of C/EBPα and terminal fat cell differentiation [33]. RUNX1t1 and C/EBPβ are both expressed in the proliferative neck zone of the normal gastric mucosa and, similar to adipogenesis, C/EBPβ is required for proliferation and inhibition of differentiation genes in this tissue. Thus, our data imply a regulatory loop between C/EBPβ and RUNX1t1 in gastric mucosa, although the detailed mechanism regulating the crosstalk and molecular genetic interactions remains to be addressed.

The connection between C/EBPβ and RUNX1t1 may also be relevant in hematopoietic malignancies involving the AML-ETO translocation product. Recently it has been shown that RUNX1 and C/EBPβ mark all hematopoietic genes in embryonic stem cells during hematopoietic commitment [38]. It is thus tempting to speculate that the fusion of RUNX1 and RUNX1t1 in the t(8;21) AML-ETO translocation may connect to early commitment events that involve C/EBPβ. In any case, we show that in gastric cancer development, high expression of C/EBPβ leads to reduction of RUNX1t1 expression and loss of RUNX1t1 may support unrestrained C/EBPβ function and repression of differentiation genes, including TFF1. Importantly, TFF1 deletion has also been found to promote oncogenesis, suggesting an important cross-regulation between C/EBPβ, RUNX1t1 and TFF1 [11].

## Electronic supplementary material

Below is the link to the electronic supplementary material.

ESM 1 (PDF 338 kb)
