# Supplementary material for: C/EBPβ regulates homeostatic and oncogenic gastric cell proliferation
Source: J Mol Med (Berl). 2016 Aug 13;94(12):1385–95. doi: 10.1007/s00109-016-1447-7 (PMC5143359; doi:10.1007/s00109-016-1447-7)
Supplement: Supplementary file 1 — (PDF 338 kb) [file 109_2016_1447_MOESM1_ESM.pdf]

Electronic supplementary material (ESM)
J Mol Med 2016

C/EBPβ regulates homeostatic and oncogenic gastric cell proliferation

Goncalo Regalo, Susann Förster, Carlos Resende, Bianca Bauer, Barbara Fleige, Wolfgang Kemmner, Peter M Schlag, Thomas F. Meyer, José C Machado, Achim Leutz

Max-Delbrueck-Center for Molecular Medicine, 13125 Berlin, Germany
Humboldt-University of Berlin, Institute of Biology, 10115 Berlin, Germany

Correspondence:
Achim Leutz
aleutz@mdc-berlin.de

Mailing address:
Max-Delbrueck-Center for Molecular Medicine
Robert-Roessle-Str.10
13125 BERLIN
Germany
Phone: +49 30 9406 3735
Fax: +49 30 9406 3298

Supplementary Table 1. List of the 20 genes with higher up-regulation score in the C/EBPβ KO stomach.

p-value	FC	Gene symbol	Gene name
1.37E-06	2.1	Crim1	cysteine rich transmembrane BMP regulator 1 (chordin like)
1.17E-05	35.3	Krtap3-2	keratin associated protein 3-2
1.33E-05	5.6	Cwh43	cell wall biogenesis 43 C-terminal homolog (S. cerevisiae)
3.74E-05	1.6	Tnip1	TNFAIP3 interacting protein 1, transcript variant 1
6.92E-05	2	Cidec	cell death-inducing DFFA-like effector c
7.80E-05	2	Pik3ip1	phosphoinositide-3-kinase interacting protein 1
9.68E-05	1.9	Unc5b	unc-5 homolog B (C. elegans)
1.21E-04	2.1	Sys1	SYS1 Golgi-localized integral membrane protein homolog (S. cerevisiae)
1.64E-04	2.3	E130012A19Rik	RIKEN cDNA E130012A19 gene
1.80E-04	2	Gm8221	predicted gene 8221, non-coding RNA
2.03E-04	2.1	Ly86	lymphocyte antigen 86 (Ly86)
2.06E-04	2.7	Muc13	mucin 13, epithelial transmembrane
2.59E-04	2.8	Adck3	aarF domain containing kinase 3, nuclear gene encoding mitochondrial protein, transcript variant 1
2.67E-04	3.5	Gkn2	gastrokine 2
2.83E-04	1.5	Procr	protein C receptor, endothelial

Supplementary Table 2. List of the 20 genes with higher downregulation score in the C/EBPβ KO mucosa

p-value	FC	Gene symbol	Gene name
2.37E-07	112.5	Cpsf3l	cleavage and polyadenylation specific factor 3-like
5.87E-07	20.5	Gltpd1	glycolipid transfer protein domain containing 1
8.00E-07	17.4	BC021614	cDNA sequence BC021614
1.45E-06	44.2	Cebpb	CCAAT/enhancer binding protein (C/EBP), beta
6.13E-05	2.2	Pusl1	pseudouridylate synthase-like 1
7.01E-05	3.6	Sftpd	surfactant associated protein D
7.37E-05	2.8	Tdh	L-threonine dehydrogenase
1.24E-04	3	Nppa	natriuretic peptide type A
1.30E-04	3.4		CYT3_MOUSE (P35175) Stefin 3, partial (93%)
1.46E-04	17.4	Defb3	defensin beta 3
1.47E-04	4.7	Slc5a5	solute carrier family 5 (sodium iodide symporter), member 5
2.31E-04	1.8	Fam132a	family with sequence similarity 132, member A (Fam132a),
2.56E-04	1.9	Nr1h3	nuclear receptor subfamily 1, group H, member 3, transcript variant 1,
2.71E-04	1.6	Larp1	La ribonucleoprotein domain family, member 1
2.73E-04	2.2	Flrt1	fibronectin leucine rich transmembrane protein 1

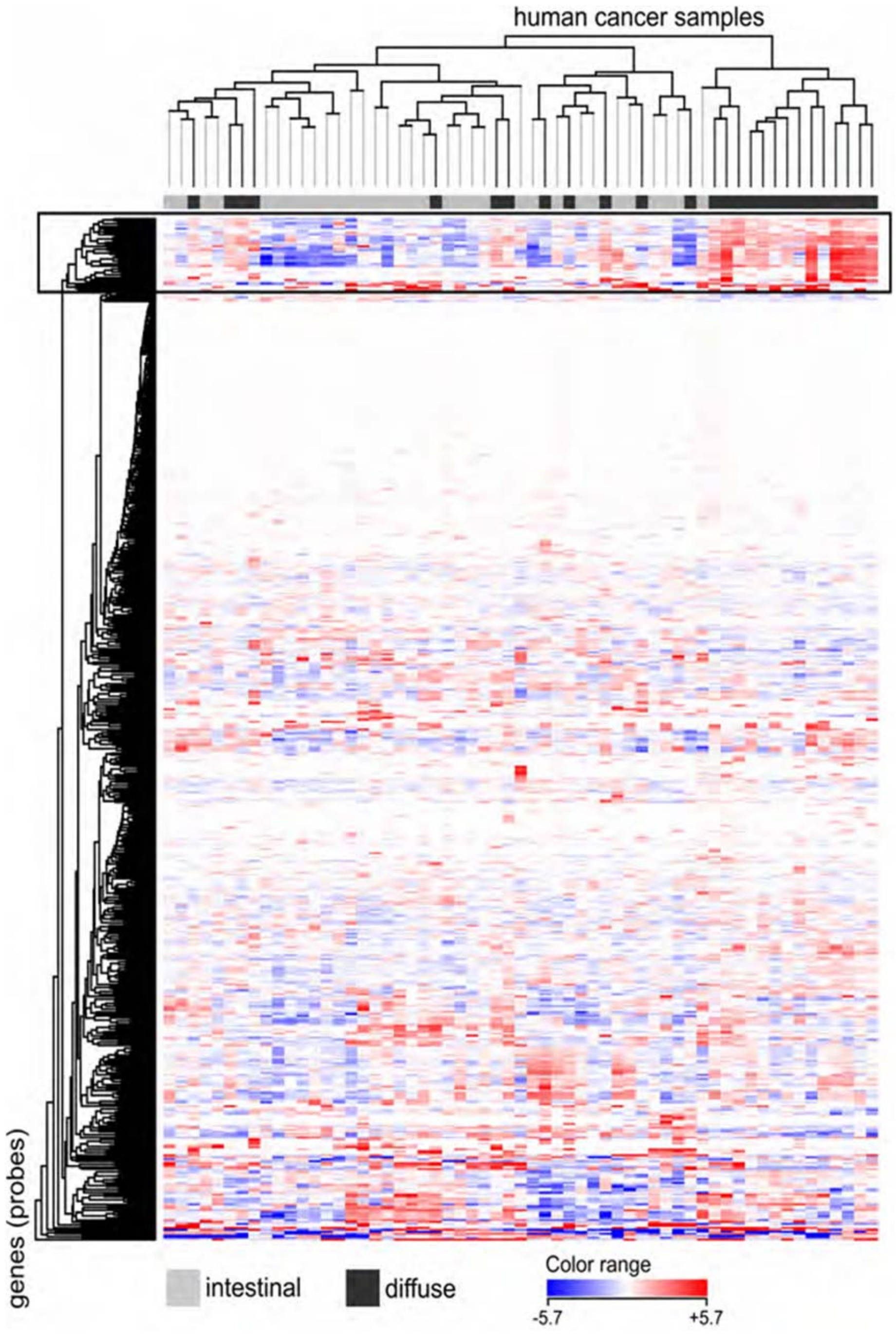

Supplementary Figure 1. Heat map originated by the comparison of mouse microarray data displaying differences between C/EBPβ KO and WT mouse stomach, and a set of human gastric cancer samples. The majority of murine genes were not regulated in human gastric cancer (whitish spots in the map). However, one cluster of genes showed explicitly strong regulation (highlighted). The members of this strongly regulated gene cluster showed down-regulation in the intestinal tumors compared to the diffuse-type ones with differences ranging between 1.8 and 6.2 with and overall classification correctness for histological type of 78%.

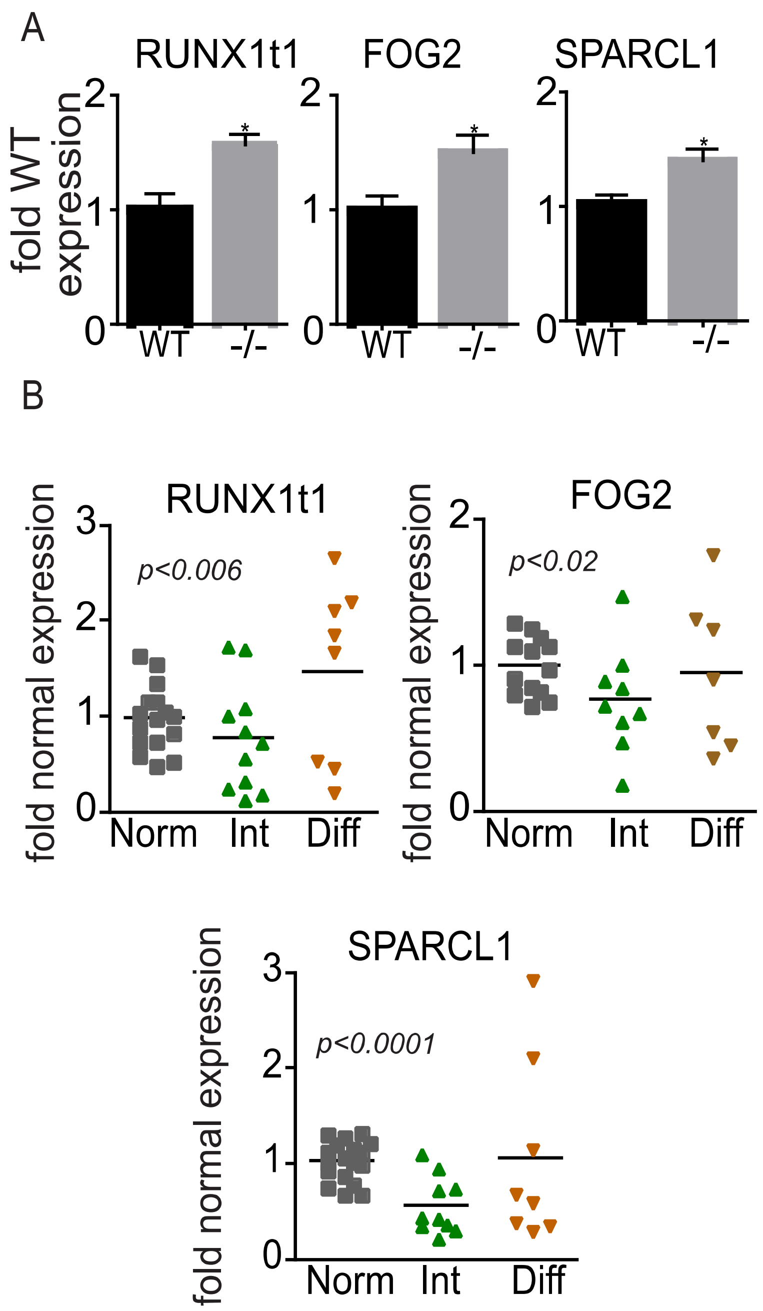

Supplementary Figure 2. Confirmation of the microarray expression analysis results. A) Expression evaluation of FOG2, SPARCL1 and RUNX1t1 expression in wildtype (WT) and C/EBPβ KO stomach (5 animals/group) by qPCR, showing upregulation in the C/EBPβ KO. B) RNA expression analysis of FOG2, SPARCL1 and RUNX1t1 in intestinal and diffuse gastric cancer cases as determined by real-time PCR. Tumor vs. normal ratios were established for each case. Values above 1 entail upregulation, whereas expression bellow 1 refers to downregulation. Visible is the decreased expression of the gene set in intestinal type gastric cancer cases in comparison to normal. Significance displayed in graphic refers to Normal vs. Intestinal-type comparison.

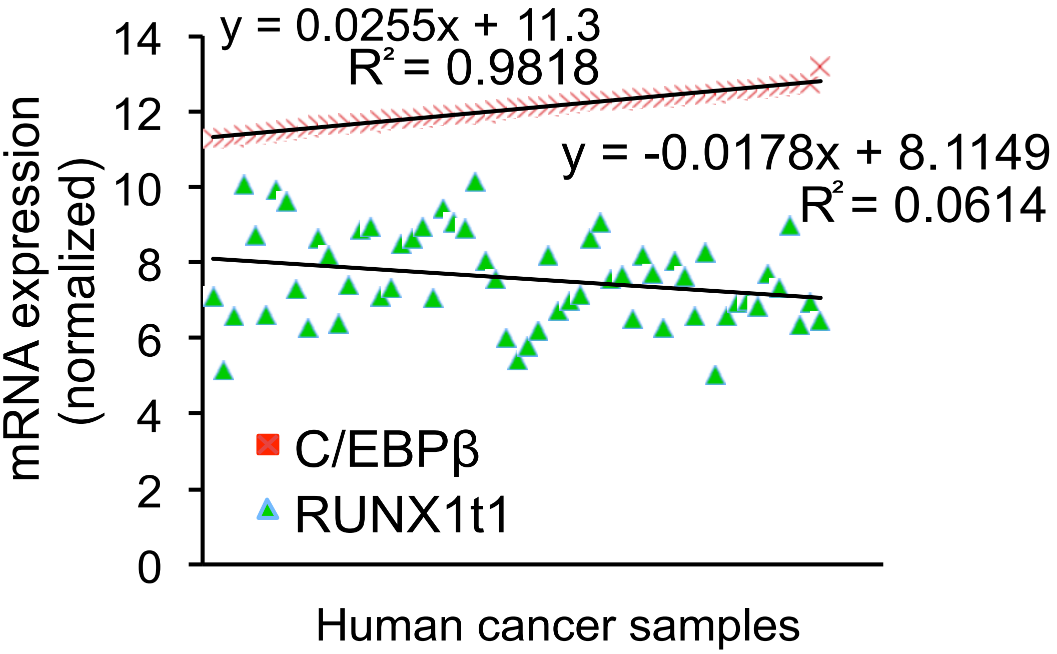

Supplementary Figure 3. mRNA expression of C/EBPβ and RUNX1t1 in 59 human gastric cancer samples. Normalized mRNA expression values were obtained from microarray data with no baseline transformation to allow easy visualization of the semi-quantitative expression values. The cancer samples were sorted according to ascending CEBPβ expression values. Trendlines were added using linear regression, and P-value and R-squared value (square of the correlation coefficient) were calculated accordingly.

Supplementary Experimental Procedures

Supplemental Table 3. List of primers used in quantitative real-time PCR analysis of gene expression.

Gene	Species	Primer (F/R) 5'-3'
C/EBPβ	Human	gacaagcacagcgacgagta
		agctgctccaccttcttctg
FOG2	Human	tggggacacacagtcagaga
		cctcagagatggccttcgta
FOG2	Mouse	tgggatggaccaggagag
		gacgagctcttcaccctctg
SPARCL1	Human	agagcaccaagaggccaag
		ctctcatccgtagaggaaactga
SPARCL1	Mouse	tcctgcttgtacggactttg
		ttccttcaaggtgatgtgctt
RUNX1t1	Human	ccctcgctagacgtgaactc
		tgctgtttggtaaagcatcg
RUNX1t1	Mouse	agttcgcacccttgt

		ttcgtgctgagcgag
Ki67	Mouse	ccacactgtgtcgtcgtttg
		ccgtgcgcttatccattca
PCNA	Mouse	cgaagcaccaaatcaagaga
		cggcatatacgtgcaaattc
MUC6	Mouse	cctctgctgcgactgtctaa
		tgggagtgggaagataatgg
MUC5ac	Mouse	ctgtggagcatggggaaat
		gaaccacagacctgctccac
Cyclin A1	Mouse	gctaccttccagaagctgaagt
		cagggtctctgtgcgaagtt
Cyclin E1	Mouse	gcagcgagcaggagacaga
		gctgcttccacaccactgtctt
Cyclin D3	Mouse	tgccaaaacgccccagtac
		cgggatgcccgaagga
P15	Mouse	agatcccaacgccctgaac

		cccatcatcatgacctggatt
